# Peak match sprinting speed during soccer matches: analysing the pre- and post-peak speed dynamics

**DOI:** 10.5114/biolsport.2024.136089

**Published:** 2024-08-08

**Authors:** Hugo Silva, Fábio Yuzo Nakamura, Ghazi Racil, Antonio Gómez-Diaz, Pedro Menezes, Karim Chamari, Rui Marcelino

**Affiliations:** 1 Research Center in Sports Sciences, Health Sciences and Human Development, CIDESD, CreativeLab Research Community, Vila Real, Portugal; 2 University of Maia, Maia, Portugal; 3 Research Unit (LR 23JS01) “Sport Performance, Health & Society”; Higher Institute of Sport and Physical Education of Ksar Said; University of Manouba, Tunis, Tunisia; 4 Clube de Regatas do Flamengo, Rio de Janeiro, Brazil; 5 Spanish Football Federation (RFEF), Madrid, Spain; 6 Naufar, Wellness and Recovery Center, Doha, Qatar; 7 Portugal Football School, Portuguese Football Federation, Oeiras, Portugal

**Keywords:** Acceleration, Deceleration, Football, Match analysis, Running

## Abstract

This study aimed to characterize match peak speeds, during a 20-second time window (10 seconds immediately before and after the match peak speed), in soccer matches. Twenty elite soccer players were monitored with global navigation satellite system (GNSS) devices during six soccer matches from the Brazilian first division. After identifying the peak speed, individual speeds within 10 seconds before and 10 seconds after were collected at each 0.1-second interval (10 Hz). Intra-individual speed differences were assessed at every second of the selected window using paired mean differences (with effect sizes and 95% confidence intervals). Match peak speeds ranged from 29.11 km/h to 31.64 km/h. Speeds registered 10 seconds before and 10 seconds after the match peak speed ranged from 5.11 km/h to 9.21 km/h and 6.90 km/h to 7.65 km/h, respectively. Speed increased moderately (effect size [95% confidence interval]: 0.68 [0.64, 0.73]) 4 seconds before the match peak speed and decreased moderately (effect size [95% confidence interval]: -0.73 [-0.78, -0.68]) 3 seconds after the maximal effort. Match peak speeds were achieved from flying starts, differing from the current sprint test procedures. Players needed to accelerate from speeds of approximately 10 km/h to reach their peak speed. After the match peak speed, players decelerated faster than they accelerated but without reaching a complete stop. Nevertheless, preparing players for intense decelerations should not be disregard. Players can benefit from field tests and training sessions that provide similarities to what occurs during competition, and accelerations should also be considered when referring to peak speeds.

## INTRODUCTION

Soccer players are often tested to assess individual strengths and weaknesses and to evaluate the effectiveness of training programmes [1]. The sprint test is one of these tests and is used to assess players’ ability to quickly accelerate and achieve/maintain a maximal speed in 10–40 m distances [2]. Usually, players are assessed with timing gates and/or global navigation satellite systems (GNSS), with the first equipment reporting time for completing the test, and the second reporting the speed reached [2]. With tests results, practitioners identify the faster players and the ones who need to develop their speed capacities, taking into consideration their playing role and individual characteristics. However, analysing match performances with peak speeds achieved during tests is questionable as test speeds generally exceed match peak speeds [3, 4], although opposite findings were also reported [5]. For example, during matches, elite soccer players achieved 92% of their peak speed registered during sprint field tests [3]. The differences observed can probably be explained by the starting conditions of the sprint, since a high percentage (84%) of the achieved peak speed is attained in the first 10 of the 30 m standard sprint test [6]. Considering that match sprints are usually shorter than 10 m [7], a standing start would probably not allow them to reach their peak speed. Regarding this limitation, some authors adapted test protocols to increase similarity to matches. For example, players can be requested to adopt a specific speed at the starting line (flying start) [2]. Moreover, match situations, such as counter attacks, need to occur to allow players to reach maximal speeds. For instance, forwards (FW) could have more space to run as they usually face only the defence line, while center midfielders (CM) could suffer more pressure and have less space to develop high-speed actions [8]. Accordingly, Djaoui et al. [3] reported higher match peak speeds for advanced positions (wide midfielders [WM] and FW) when monitoring elite soccer players. Moreover, these offensive positions perform more sprint actions in the opponent field area, highlighting the importance of tactical roles of each playing position [9].

Within the analysis of how players reach sprint speeds during competition, Di Salvo and colleagues [7] classified sprints into two types: “explosive” (defined as those where players reached the sprint threshold [> 25.2 km/h] without entering the high-speed running category [19.8–25.2 km/h] in the previous 0.5 seconds and “leading” (defined as those where players achieved the sprint threshold while entering the high-speed running category in the previous 0.5 seconds). They found that there were more “leading” sprints during matches, which means that players gradually accelerate to reach the sprint threshold rather than performing fast accelerations. Additionally, during matches, sprint efforts are mostly sustained for short durations (< 5 seconds) [10]. This information can provide important insights to practitioners when they assess competition peak efforts.

However, previous research has focused the analysis on the distance covered above a given sprint threshold, while little is known on how players achieve match peak speeds [11]. For instance, considering the sprint test characteristics, one may question the validity of standing starts, as a testing procedure, since sprint performances are significantly influenced by the leading distance (distance before the initial timing gates) [12]. Besides the required validity and reliability, a test can also benefit from a more ecological approach, improving the usefulness of test results to practitioners.

By understanding how players achieved their match peak speed, practitioners can develop training strategies or test procedures to improve and assess sprint ability in soccer players. Furthermore, game success can even increase, as sprints are closely related to goal situations [13]. Additionally, considering that maximum speeds can subject hamstrings to extremely high loads [14], it may not be surprising that both hamstring injuries [15] and sprint distance have increased in recent years [16]. However, even if sprints increase the risk of hamstring injuries, the progressive exposure of players to these intense efforts can provide an important protective effect [17]. Therefore, the purpose of this study is to analyse how players reach and leave their match peak speed, by identifying speed changes during a 20-second time window (10 seconds immediately before and after the match peak speed).

## MATERIALS AND METHODS

### Study Design

During this observational study, a cohort of professional soccer players from a highly ranked Brazilian team was subjected to monitoring over the course of six consecutive national first division matches during the 2022 season. Subsequent to the acquisition of raw data from the players, a comprehensive analysis was performed in order to establish match peak speeds.

### Subjects

This study included twenty male elite players with an average age of 27.8 ± 5.3 years, height of 179.4 ± 5.9 cm and body mass of 73.3 ± 6.5 kg. These players were monitored during competition and were divided (by coaching staff) into the following playing positions: 4 central defenders (CD), 3 fullbacks (FB), 5 central midfielders (CM), 5 wide midfielders (WM) and 3 forwards (FW). From the six matches, 90 observations were collected. Goalkeepers were excluded from this study due to the particularity of the position. Ethics Committee clearance was obtained by the University Institute of Maia (35/2021) and the study was conducted in accordance with the Declaration of Helsinki. Match data were collected as a standard procedure of the club, and without any interference from the research team [18].

### Procedures

Players were monitored with a 10 Hz GNSS (WIMU Pro – Realtrack Systems). This device was certified by FIFA (certification number: 1004497) and previously validated [19]. Devices were secured between the upper scapulae, at approximately the T3-4 junction, and were activated 15 minutes before use, in accordance with the manufacturer’s instructions. To avoid interunit error, each player used the same WIMU device throughout the data collection period.

Competition raw data were retrieved from the GPS software (WIMU SPRO) as speed (km/h) and time (milliseconds). By analysing raw data, which were not treated by the equipment software, filters were created to minimize potential noise: first, speeds above 44.45 km/h were excluded [20]; second, the players’ highest speed achieved during each match was recorded as the match peak speed, provided that another effort within 1 km/h slower than the peak speed occurred during the same match (e.g., 32.7 km/h would be a valid peak speed if another effort was registered at a speed ≥ 31.7 km/h), and if the match peak speed was superior to the sprint threshold (> 25.2 km/h). For each player during each match, speeds were collected at 0.1-second intervals (10 Hz: 10 samples per second [21]) from 10 seconds before and 10 seconds after the match peak speed. For example, if player A participated in 5 matches, then that player would have 5 peak match speeds with the respective 20-second window.

### Statistical Analysis

Mean ± standard deviation (SD) was calculated for speeds at each 0.1-second interval, according to the playing position and for all the players. Speed differences during the selected timeline were compared at every second of the 20-second window using paired mean differences, which were estimated in jamovi with the ESCI package [22, 23]. An intraindividual comparison was performed by comparing the individual speeds registered at one-second intervals (consecutive intervals). These differences were calculated for each second within the timeline (9–10 seconds before, 8–9 seconds before, and so on, until 9–10 seconds after the maximal effort). This procedure was repeated for each player match peak speed. As an example, the registered speed of player A during the 8-second interval was compared with the registered speed of player A during the 9-second interval. Cohen’s *d* effect sizes were established as trivial (< 0.2), small (0.2 < 0.6), moderate (0.6 < 1.2), large (1.2 < 2.0), very large (2.0 < 4.0) and extremely large (> 4.0) with 95% confidence intervals. If the confidence interval crossed zero, the effect size was considered unclear [24]. Match peak speed differences between playing positions were analysed using independent mean differences, which were estimated using jamovi with the ESCI package [22, 23]. Cohen’s *d* effect sizes were calculated and categorized in the same way as described above.

## RESULTS

Match peak speeds ranged from 29.11 km/h to 31.64 km/h, and speeds registered 10 seconds before and 10 seconds after the registered peak speed ranged from 5.11 km/h to 9.21 km/h and 6.90 km/h to 7.65 km/h, respectively (Table 1).

**TABLE 1:** Mean ± SD speeds (km/h) for each 1 second interval and for peak speeds registered according to playing positions (*n* = number of match peak speeds).

Timeline (seconds)	CD *n* = 20	FB *n* = 10	CM *n* = 19	WM *n* = 24	FW *n* = 17
*-10*	5.11 ± 3.60	7.40 ± 4.09	8.02 ± 4.94	8.30 ± 5.43	9.21 ± 6.84
*-9*	5.53 ± 4.34	8.78 ± 6.02	8.54 ± 4.89	8.42 ± 6.87	7.96 ± 5.34
*-8*	7.07 ± 5.08	10.03 ± 7.58	9.37 ± 4.71	9.18 ± 6.94	8.88 ± 5.39
*-7*	8.18 ± 5.10	11.24 ± 7.65	10.39 ± 5.15	9.47 ± 6.19	8.99 ± 5.18
*-6*	9.35 ± 5.95	13.08 ± 8.57	12.59 ± 5.12	10.05 ± 6.30	8.78 ± 5.10
*-5*	11.99 ± 5.69	15.07 ± 9.04	16.69 ± 4.70	12.28 ± 6.66	9.45 ± 5.55
*-4*	14.59 ± 7.21	18.93 ± 7.86	18.49 ± 5.10	16.40 ± 6.45	12.70 ± 5.83
*-3*	18.02 ± 6.54	22.53 ± 5.32	21.20 ± 5.19	21.48 ± 4.97	18.85 ± 4.59
*-2*	22.48 ± 5.50	26.57 ± 3.80	24.54 ± 4.08	25.43 ± 3.27	24.03 ± 2.74
*-1*	25.75 ± 5.08	29.29 ± 3.15	27.43 ± 2.95	28.67 ± 2.46	27.38 ± 2.39

*Match Peak Speed*	*29.11 ± 1.64*	*31.64 ± 1.94*	*30.26 ± 2.23*	*30.68 ± 2.61*	*29.83 ± 1.88*
*+1*	25.34 ± 5.61	27.83 ± 4.97	27.68 ± 3.08	26.82 ± 5.47	26.72 ± 3.62
*+2*	20.14 ± 6.55	20.85 ± 8.14	21.97 ± 6.63	19.12 ± 8.91	20.02 ± 7.14
*+3*	14.91 ± 7.63	13.74 ± 7.92	15.05 ± 7.54	13.23 ± 9.56	14.78 ± 6.78
*+4*	12.55 ± 7.19	11.56 ± 6.55	12.27 ± 6.72	10.46 ± 8.94	10.77 ± 5.21
*+5*	11.07 ± 6.11	11.37 ± 5.72	9.80 ± 6.00	10.13 ± 8.20	9.39 ± 5.22
*+6*	8.74 ± 5.84	10.66 ± 4.26	9.51 ± 5.11	9.91 ± 6.60	8.74 ± 5.13
*+7*	7.63 ± 5.50	8.83 ± 5.40	9.15 ± 4.60	9.40 ± 5.66	8.28 ± 4.48
*+8*	7.59 ± 5.41	7.92 ± 5.69	7.88 ± 4.24	8.39 ± 5.38	7.71 ± 3.56
*+9*	7.47 ± 5.09	7.65 ± 6.19	6.90 ± 4.11	7.46 ± 5.75	7.41 ± 3.61
*+10*	7.34 ± 4.62	7.45 ± 6.31	6.58 ± 4.23	7.01 ± 5.11	6.55 ± 3.01

CD = Central defenders; FB = Fullbacks; CM = Central midfielders; WM = Wide midfielders; FW = Forwards.

**FIG. 1:**
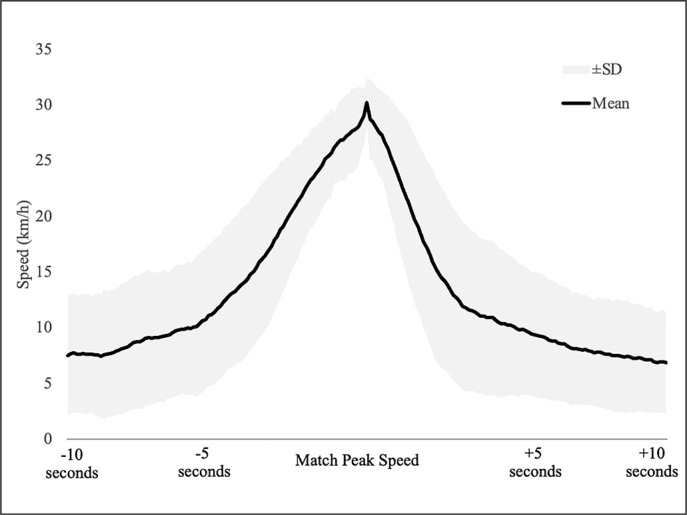
Individual registered speeds (km/h) during the 20-second window (10 seconds before and 10 seconds after the match peak speed). Speed variance decreases (smaller SD) near the match peak speed.

Across the collected data, greater speed variations were registered further away from the match peak speed, as seen by the SD presented in Figure 1. That is, when players approached the peak effort, speed variations diminished. Five seconds before and 5 seconds after the match peak speed, speeds ranged between 5 and 10 km/h (Figure 1).

**FIG. 2:**
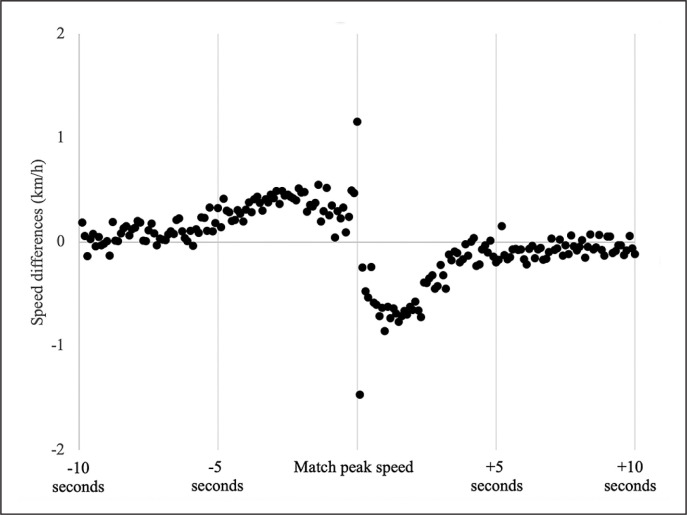
Speed changes for every 0.1 second within the 20-second timeline (10 seconds before and after the match peak speed). Speed changes near 0 km/h probably represent speed fluctuations, which occurred mainly between 10 and 5 seconds before and 5 and 10 seconds after the match peak speed. Speed changes above 1 km/h occurred only immediately before (acceleration) and after (deceleration) the match peak speed.

Speed increased (acceleration) moderately 4 seconds before the match peak speed and decreased (deceleration) moderately 3 seconds after the maximal effort (Table 2), highlighting that players decrease their speed faster than they increase it. Specifically, the largest speed increase was found between 4 and 3 seconds before the peak effort (+ 4.25 km/h), while the largest speed decrease was found between 1 and 2 seconds after the peak effort (-6.55 km/h) (Table 2). Additionally, increases (by accelerating) and decreases (by decelerating) in players’ speed were > 1 km/h immediately before (0.1 s) and after (0.1 s) the match peak speed, respectively (Figure 2). Differences between positions’ peak speeds are presented in Table 3.

**TABLE 2:** Mean paired differences (95% Confidence Intervals [CI]) with effect sizes (95% CI) of speed differences between 1 second consecutive intervals.

Timeline (seconds)	Mean paired differences (95% CI)	Effect Size (95% CI)	Effect Size
*10–9*	0.15 (-0.10, 0.40)	0.03 (-0.02, 0.07)	*Unclear*
*9–8*	1.03 (0.83, 1.24)	0.18 (0.14, 0.21)	*Trivial*
*8–7*	0.69 (0.46, 0.93)	0.12 (0.08, 0.16)	*Trivial*
*7–6*	1.04 (0.76, 1.32)	0.17 (0.13, 0.22)	*Trivial*
*6–5*	2.40 (2.12, 2.67)	0.37 (0.33, 0.41)	*Small*
*5–4*	3.10 (2.80, 3.40)	0.46 (0.41, 0.51)	*Small*
*4–3*	4.25 (3.99, 4.52)	0.68 (0.64, 0.73)	*Moderate*
*3–2*	4.18 (3.97, 4.38)	0.84 (0.80, 0.88)	*Moderate*
*2–1*	3.13 (2.99, 3.28)	0.80 (0.76, 0.85)	*Moderate*

**Match Peak Speed**			
*1–2*	-6.55 (-6.89, -6.21)	-1.04 (-1.10, -0.98)	*Moderate*
*2–3*	-5.72 (-6.02, -5.41)	-0.73 (-0.78, -0.68)	*Moderate*
*3–4*	-2.93 (-3.24, -2.62)	-0.38 (-0.42, -0.34)	*Small*
*4–5*	-1.06 (-1.31, -0.81)	-0.15 (-0.19, -0.12)	*Trivial*
*5–6*	-0.95 (-1.17, -0.73)	-0.16 (-0.19, -0.12)	*Trivial*
*6–7*	-0.97 (-1.17, -0.76)	-0.18 (-0.22, -0.14)	*Trivial*
*7–8*	-0.65 (-0.84, -0.47)	-0.13 (-0.17, -0.09)	*Trivial*
*8–9*	-0.43 (-0.59, -0.27)	-0.09 (-0.12, -0.06)	*Trivial*
*9–10*	-0.37 (-0.51, -0.24)	-0.08 (-0.11, -0.05)	*Trivial*

Note: negative values indicate speed decrease between intervals.

## DISCUSSION

The aim of this study was to analyse what happens in terms of speed variation before and after a player reaches individual peak match speeds. With this novel 20-second analysis, we can identify when speed most increases, when speed most decreases, and at what speed the effort starts and ends. The main findings of this study were that match peak speeds were generally reached with progressive speed increases and not with explosive accelerations; speed increases and decreases were most notable within 4 seconds before and 3 seconds after the peak effort; and finally, players start and end those efforts from flying starts and not stationary positions.

The replacement of sprint field tests for competition monitoring (with GPS equipment) has already been proposed to assess the peak speed [5]. Furthermore, this is an interesting discussion regarding sprint thresholds, as relative thresholds (calculated as a percentage of the peak speed registered during tests or during competition) can be a more appropriate strategy to monitor sprint events [11, 25]. Our study contributes to that debate, by showing that during competition, players do not replicate test procedures, since they start their peak effort from flying starts instead of stationary starts. As so, if practitioners intend to use field tests to record a reference value to apply relative thresholds, adaptations should be implemented, such as flying starts [2].

However, we can discuss whether match peak speeds truly represent players’ capacities. Since matches elicit short and intense efforts from players, reaching speeds higher than those obtained in > 20 m sprinting tests would be difficult [26, 27]. In our study, we assessed match peak speeds achieved during six competitive matches, which represent players’ maximal values for competition and not their absolute maximal values, as maximal speeds registered during matches are usually equivalent to 90% of players’ maximal speeds registered during field tests [3, 27–29]. This means that match peak speed can be seen as representative of players’ capacities within a specific context (competition). The match context will also influence the peak speeds of different playing positions. In our study, mean peak speeds ranged from 29.11 km/h (CD) to 31.64 km/h (FB), with wide positions reaching higher values (Table 3), while previous research reported higher values for FW during matches [3, 29, 30]. These differences could be explained by the match variability, since sprint actions occur within different phases of the match [31], and by the required responses to different technical-tactical demands of the game [32].

**TABLE 3:** Independent mean differences with 95% Confidence Intervals (CI) (with effect sizes [ES] and 95% CI) between playing positions.

	CD	FB	CM	WM	FW
**CD** (n = 20)	-	2.50 [1.12, 3.88] ES: 1.40 [0.63, 2.40]^L^	1.13 [-0.14, 2.39] ES: 0.57 [-0.06, 1.26]^U^	1.55 [0.19, 2.93] ES: 0.69 [0.09, 1.35]^M^	0.69 [-0.48, 1.87] ES: 0.39 [-0.26, 1.09]^U^

**FB** (n = 10)		-	-1.38 [-3.09, 0.34] ES: -0.62 [-1.50, 0.14]^U^	-0.95 [-2.82, 0.92] ES: -0.38 [-1.18, 0.37]^U^	-1.81 [-3.37, -0.25] ES: -0.92 [-1.87, -0.14]^M^

**CM** (n = 19)			-	0.42 [-1.10, 1.95 ES: 0.17 [-0.44, 0.80]^U^	-0.44 [-1.84, 0.97] ES: -0.21 [-0.90, 0.46]^U^

**WM** (n = 24)				-	-0.86 [-2.36, 0.64] ES: -0.36 [-1.03, 0.27]^U^

**FW** (n = 17)					-

Note: CD = Central defenders; FB = Fullbacks; CM = Central midfielders; WM = Wide midfielders; FW = Forwards. L = large effect size; M = moderate effect size; U = unclear effect size. When the difference (or ES) is negative, the highest speed is relative to the position identified on the first column.

Our findings also relate to previous research that focused on acceleration efforts. First, as illustrated by Osgnach et al. [33], an acceleration starting from 9 km/h can double or triple the metabolic power compared to running constantly at that specific speed. This speed example is similar to what we found at 5 seconds before players accelerate to their match peak speed. Hence, we should consider the high metabolic power needed to reach this maximal effort. Second, the acceleration capacity decreases as the starting speed increases [34, 35]. This can raise questions of how players are able to achieve their maximal speed from relatively high starting speeds. However, according to the study of Morin et al. [34], a player would be capable of performing a maximal acceleration close to 5 m/s^2^ from a starting speed of 10 km/h (2.8 m/s). We did not assess acceleration and deceleration magnitudes, but speed changes were higher near the match peak speed, which could mean that players’ speed probably fluctuates until 5 seconds prior to their match peak speed, when players increase their speed (Figure 3). During this short period (4 seconds before and 3 seconds after), players increased (before) and decreased (after) their speed by ~ 10 km/h and ~ 16 km/h, respectively (Table 1). This probably means that a particular match situation elicited a reaction from players around 4 seconds before they reached their match peak speed (Table 2). Similarly, since no sudden stop was reported (on average) following match peak speeds, players were probably not required to perform a sudden, sharp-angled change of direction [36]. This is an important consideration, as intense decelerations expose players to higher hamstring and knee (anterior-cruciate ligament) injury risk [37]. Furthermore, even if near-maximal speed efforts are rare during matches [4], avoiding sudden increases can help players manage the fatigue and muscle damage associated with these efforts [38].

Additionally, when analysing how players reached their match peak speed, we can highlight the importance of acceleration, which differs from sprint efforts regarding muscles’ activation. Specifically, previous research has reported that during the early stance of the acceleration sprint, the activation of the biceps femoris – the most affected muscle in hamstring injuries [39] – was greater than the activation of the semitendinosus muscle [40]. Interestingly, this difference was not reported during maximum speed sprint. This underlines that the peak speed effort comprises a significant physiological effort (acceleration) that can impact the players’ hamstring injury risk.

### Limitations and future research

Our study is limited by using only one team and the lack of analysis of other contextual factors. For example, match location (home or away) and outcome (win, draw or loss) affect physical demands registered in soccer players [41], and we have not included these situations in our study. Nevertheless, this study highlights the past and the future (before and after) of match peak speeds, for a large timeline period (20 seconds), which can provide new perspectives for future studies involving different and larger cohorts. Future research could compare peak speed data obtained from GNSS devices with video analysis.

## CONCLUSIONS

Our study presented a novel approach regarding match peak speed, analysing those efforts during a prolonged time window (20 seconds). With this analysis, we saw each speed registered within the 20-second window, allowing us to identify how and when players reach and leave their peak speed. We found that match peak speeds were achieved from flying starts, instead of stationary starts, which differs from the most common sprint test procedures. This can elicit higher metabolic power demands from players due to accelerations starting at speeds ~10 km/h. After the match peak speed, players decelerated faster than they accelerated but without reaching a complete stop. Although an encouraging finding – with players avoiding very intense decelerations – the continuous exposure of players to intense decelerations is important, especially considering the risks involved with those actions. Finally, how players reach match peak speeds during matches should be replicated during training sessions, which can be achieved by proposing sprint training with flying starts.

### Practical implications

Considering our findings, four different practical applications should be considered: field tests, training sessions, physiological demands, and playing positions. First, and regarding field tests, practitioners should avoid analysing sprints in the match while considering field test performances; or they should adapt their test, allowing players to start sprints with flying starts, and allowing short times to achieve their maximal speeds. The reasoning for this is based on the differences in how players reach peak speeds in both activities (tests and matches). Second, and regarding training sessions, sprint training designed for soccer players should incorporate such flying sprints in addition to explosive sprints. According to our findings, the ability to accelerate from flying starts may represent higher importance to reach match peak speeds than the ability to accelerate from static starts, which is in line with previous research that suggested flying sprints to develop maximal speeds [42]. One additional strategy to improve accelerations from flying starts is vertical-oriented plyometrics, which was identified as more appropriate than horizontal-oriented plyometrics to develop accelerations between 10 and 20 m [43]. Thirdly, the demands associated with these peak efforts should also be considered. In this topic, we should divide accelerations from decelerations. As seen in Figure 1, players sprinted (> 25 km/h) for short periods, which was also highlighted in previous studies [10]. This means that we should pay close attention to speed increases (accelerations) and decreases (decelerations). Considering the higher metabolic power associated with accelerations [33], practitioners need to acknowledge that match peak speeds can elicit high metabolic power from players. Furthermore, even if soccer players progressively decelerate from match peak speeds, practitioners should prepare players for high-intensity decelerations, due to the potential injury risk associated with those efforts [37]. And finally, while monitoring peak efforts, practitioners should also consider playing positions as differences may be expected. These differences may occur due to individual capacities or due to match context [44, 45]. While wide positions may have more field space to achieve peak speeds, players’ capacities should be developed to ensure that players are ready for decisive match moments.
